# Clinical Impact of Postoperative Pneumonia on Ventilatory Dependency and ICU Utilization Following Adult Cardiac Surgery: Preliminary Findings From a Single-Center Observational Cohort

**DOI:** 10.7759/cureus.104957

**Published:** 2026-03-09

**Authors:** K Said, M E Mehdi, Hakim El Baraka, Najib Bouhabba, Mehdi Nabil, Hicham Kbiri, Abdelatif Chlouchi, Hamza Najout, Mourad Ababou, Amine Bentahar, Moncef Salek, Ilias Benchafai, Abdeljalil Abouchadi, Rachid Elbarni, Abdelmajid Bouzerda, Hatim A El Ghadbane, Mohammed Drissi, Bassam Bencharfa, Chakib Chouikh, Adbelatif Benbouha, Naoufal Elghoul, Hicham Janah, Hicham Sallahi, Youssef Qamouss, Ali Khatouri, Said Zouhair, Mohamed Zyani

**Affiliations:** 1 Anesthesiology and Reanimation, Mohamed V Military Teaching Hospital, Rabat, MAR; 2 Cardioanesthesia, Mohamed V Military Teaching Hospital, Rabat, MAR; 3 Anesthesiology and Reanimation, Avicenna Military Hospital, Marrakesh, MAR; 4 Anesthesiology and Perioperative Medicine, Military Hospital Oued Eddahab, Agadir, MAR; 5 Anesthesiology, Avicenna Military Hospital, Marrakesh, MAR; 6 Anesthesiology and ICU, Congenital Cardiovascular Surgery, Louis Pradel Hospital, Part of the Hospices Civils de Lyon (HCL), Lyon, FRA; 7 Anesthesiology, Mohamed V Military Teaching Hospital, Rabat, MAR; 8 Anesthesiology and ICU, Mohamed V Military Teaching Hospital, Mohammed V University, Rabat, MAR; 9 Cardiothoracic ICU, Marie-Lannelongue Hospital, Paris, FRA; 10 Radiology, Moulay Ismail Military Hospital, Meknes, MAR; 11 Diagnostic and Interventional Radiology, Moulay Ismail Military Hospital, Meknes, MAR; 12 Otolaryngology - Head and Neck Surgery, Avicenna Military Hospital, Marrakesh, MAR; 13 Plastic Surgery, Avicenna Military Hospital, Marrakesh, MAR; 14 General Surgery, Avicenna Military Hospital, Marrakesh, MAR; 15 Cardiology, Avicenna Military Hospital, Marrakesh, MAR; 16 Cardiovascular Anesthesiology and ICU, Mohamed V Military Teaching Hospital, Rabat, MAR; 17 Anaesthesiology, Avicenna Military Hospital, Marrakesh, MAR; 18 Anesthesiology and Critical Care, Military Hospital of Rabat, Rabat, MAR; 19 Trauma and Orthopedics, Faculty of Medicine, University Hassan II Casablanca, Casablanca, MAR; 20 Orthopedic Surgery and Traumatology, Faculty of Medicine and Pharmacy, Mohamed V Military Teaching Hospital, Rabat, MAR; 21 Pneumology, Avicenna Military Hospital, Marrakesh, MAR; 22 Orthopaedics and Traumatology, Avicenna Military Hospital, Marrakesh, MAR; 23 Anesthesia and Critical Care, Avicenna Military Hospital, Marrakesh, MAR; 24 Microbiology, Avicenna Military Hospital, Marrakesh, MAR; 25 Internal Medicine, Ibn Sina Military Hospital, Marrakesh, MAR

**Keywords:** cardiac surgery, cardiopulmonary bypass, critical care utilization, intensive care unit length of stay, mechanical ventilation, observational cohort study, perioperative risk factors, postoperative pneumonia, pulmonary morbidity, respiratory complications

## Abstract

Background

Postoperative pneumonia remains a frequent and clinically consequential complication following adult cardiac surgery with cardiopulmonary bypass (CPB), contributing to prolonged ventilatory dependency and increased intensive care resource utilization. Identification of perioperative determinants may inform targeted prevention strategies.

Methods

We conducted a single-center retrospective observational cohort study including 50 consecutive adults undergoing cardiac surgery with CPB between January 2022 and December 2023. Postoperative pneumonia occurring within seven postoperative days was defined using combined clinical, radiologic, and microbiological criteria. Baseline characteristics, intraoperative variables, and early postoperative outcomes were compared between patients with and without pneumonia.

Results

Postoperative pneumonia developed in 11 of 50 patients (22%). Patients with pneumonia more frequently required prolonged mechanical ventilation (>24 hours) compared with those without pneumonia (64% vs 23%) and had higher reintubation rates (27% vs 5%). Intensive care unit length of stay was substantially longer among pneumonia patients (mean±SD, 7.8±3.1 vs 3.9±1.7 days). Cardiopulmonary bypass duration was longer, and diabetes mellitus was more prevalent in patients who developed pneumonia.

Conclusions

In this real-world cohort, postoperative pneumonia was common and consistently associated with sustained ventilatory dependence and increased ICU utilization. These findings underscore the clinical and resource burden of postoperative pulmonary complications and support risk-stratified perioperative strategies emphasizing early extubation readiness, pulmonary-protective management, and structured postoperative surveillance.

## Introduction

Despite major advances in surgical technique, anesthetic management, and postoperative critical care, pulmonary complications remain important determinants of early morbidity and resource utilization after adult cardiac surgery. Among these, postoperative pneumonia (POP) is particularly consequential and consistently associated with prolonged mechanical ventilation, extended intensive care unit (ICU) stay, and increased hospital costs [1-4].

The reported POP incidence ranges from 10% to 25%, reflecting differences in patient susceptibility, procedural complexity, perioperative practices, and diagnostic definitions [2,5-8]. Current evidence supports a multifactorial vulnerability model in which baseline host factors - such as chronic pulmonary disease and metabolic dysregulation - interact with procedure-related stressors, including prolonged cardiopulmonary bypass (CPB), systemic inflammatory activation, and ventilatory exposure [3,7-10].

Risk prediction models, including EuroSCORE II and the Society of Thoracic Surgeons (STS) algorithms, have refined perioperative stratification in Western cohorts and consistently identify CPB duration, ventilatory exposure, and underlying pulmonary disease as dominant predictors of postoperative respiratory morbidity [9-13]. However, their calibration and external validity in North African populations remain insufficiently characterized. In Morocco, patients frequently present with a high burden of diabetes mellitus and advanced structural heart disease, factors that may modify postoperative pulmonary vulnerability beyond that captured in the existing models.

In this study, POP was defined as a new or progressive pulmonary infiltrate on chest imaging, formally reported by attending radiologists, combined with at least one systemic criterion (fever >38°C or leukocytosis/leukopenia) and one respiratory criterion (purulent secretions or worsening oxygenation). The Clinical Pulmonary Infection Score was not systematically applied; diagnoses were based on concordant clinical and radiologic documentation confirmed by the intensive care team. The use of structured radiologic and clinical criteria was intended to enhance reproducibility within the constraints of a retrospective design.

Accordingly, this single-center observational study was structured hierarchically. The primary objective was to determine the incidence of POP in adults undergoing cardiac surgery with CPB. Secondary objectives were to evaluate associations between POP and intraoperative exposures (including CPB and cross-clamp duration) and to assess its impact on prolonged mechanical ventilation, reintubation, and ICU length of stay.

We hypothesized that prolonged CPB duration would be associated with increased POP risk and greater early postoperative ICU burden.

## Materials and methods

Study design and setting

This retrospective single-center observational cohort study was conducted in the cardiac surgery department and postoperative intensive care unit of a tertiary referral center in Morocco, serving a population characterized by a high prevalence of diabetes mellitus and advanced valvular heart disease at presentation. The retrospective design was selected to capture real-world perioperative practice and postoperative outcomes without protocol-driven intervention. Reporting was performed in accordance with the Strengthening the Reporting of Observational Studies in Epidemiology (STROBE) guidelines. All consecutive adult patients aged 18 years or older who underwent cardiac surgery requiring cardiopulmonary bypass (CPB) between January 1, 2022, and December 31, 2023, were systematically screened for eligibility.

Study population

Eligible patients included adults undergoing isolated coronary artery bypass grafting, isolated valvular surgery, or combined cardiac procedures performed with CPB and planned postoperative admission to the intensive care unit. Patients were excluded if they underwent off-pump cardiac surgery, had clinical or radiologic evidence of active pneumonia or lower respiratory tract infection prior to surgery, or had incomplete medical documentation precluding reliable ascertainment of perioperative variables or outcomes. These criteria were applied to minimize misclassification bias and enhance internal validity.

Definition of postoperative pneumonia

Postoperative pneumonia was defined as a pulmonary infectious complication occurring within seven postoperative days. Diagnosis required the presence of a new or progressive pulmonary infiltrate on chest radiography or computed tomography, as formally interpreted and documented by board-certified attending radiologists, in conjunction with predefined clinical criteria.

Clinical criteria required at least one systemic sign (temperature >38°C or <36°C, leukocytosis >12,000/mm³, or leukopenia <4,000/mm³) and one respiratory criterion (purulent tracheobronchial secretions or worsening oxygenation not otherwise explained by cardiogenic or atelectatic causes).

Microbiological confirmation from respiratory specimens, including endotracheal aspirate or bronchoalveolar lavage, was incorporated when available. In cases without microbiological documentation, diagnosis required concordant radiologic findings and documented clinical deterioration prompting initiation of targeted antimicrobial therapy.

The Clinical Pulmonary Infection Score (CPIS) was not systematically applied during the study period. Given the retrospective design, formal inter-observer variability assessment for radiologic interpretation was not performed.

Data collection and variables

Demographic characteristics, baseline comorbidities, intraoperative variables, and postoperative outcomes were retrospectively extracted from electronic medical records and anesthesia information systems using a standardized data abstraction framework. Baseline variables included age, sex, body mass index, smoking history, diabetes mellitus, and chronic obstructive pulmonary disease. Although diabetes and chronic obstructive pulmonary disease were recorded as binary comorbidities, granular indices of preoperative disease optimization, such as glycated hemoglobin (HbA1c) levels or spirometric severity parameters, were not consistently available in the medical record and were therefore not incorporated into the analytic dataset. Intraoperative variables included the type of surgical procedure, total CPB duration, and aortic cross-clamp time, serving as surrogate markers of procedural complexity, ischemic burden, and systemic inflammatory exposure. Postoperative variables included duration of mechanical ventilation, requirement for reintubation, intensive care unit length of stay, and in-hospital mortality.

Data were extracted using a standardized abstraction template by trained investigators familiar with perioperative documentation practices. In cases of ambiguous or incomplete documentation, records were reviewed for internal consistency prior to data entry. Missing data were handled using complete-case analysis; variables not consistently available in the record, including detailed transfusion burden and preoperative disease-control indices, were not included in inferential analyses.

Perioperative management

Standard perioperative antibiotic prophylaxis consisted of intravenous cefazolin administered prior to surgical incision and continued for 24-48 hours postoperatively unless modified based on clinical indication or microbiological findings.

Intraoperative mechanical ventilation was performed using volume-controlled ventilation with tidal volumes typically ranging between 6 and 8 mL/kg predicted body weight and positive end-expiratory pressure (PEEP) of 4-6 cmH₂O in accordance with institutional practice.

Postoperative ventilatory management followed a lung-protective strategy targeting tidal volumes of approximately 6 mL/kg predicted body weight with individualized PEEP adjustments based on oxygenation and hemodynamic tolerance.

Extubation readiness was assessed by the intensive care team based on hemodynamic stability, adequate oxygenation defined as PaO₂/FiO₂ greater than 200, normothermia, satisfactory neurologic status, and acceptable arterial blood gas parameters. Respiratory sampling for suspected infection was performed at the discretion of the treating ICU physician when clinical deterioration occurred.

Outcomes

The primary outcome was the incidence of postoperative pneumonia within seven days following surgery. Secondary outcomes were selected to capture clinically meaningful indicators of postoperative respiratory morbidity and healthcare resource utilization and included prolonged mechanical ventilation defined a priori as ventilation exceeding 24 hours, reintubation, intensive care unit length of stay, and in-hospital mortality.

Statistical analysis

All consecutive eligible patients during the study period were included and, therefore, no a priori sample size calculation was performed. Postoperative pneumonia occurred in 11 of 50 patients corresponding to an incidence of 22.0%. The exact Clopper-Pearson 95% confidence interval for this incidence was 11.53-35.96%, reflecting expected binomial dispersion in a modest sample. Using a two-sided exact binomial framework with an alpha level of 0.05 and assuming a reference incidence of 10%, a sample size of 50 provided 68.7% power to detect an incidence of 22%. While these findings support reasonable precision for incidence estimation, the limited number of events restricts statistical power for rare secondary outcomes.

Continuous variables were summarized as mean with standard deviation after assessment of distributional characteristics. Between-group comparisons were performed using the Student's t-test for normally distributed variables or the Mann-Whitney U test when distributional assumptions were not satisfied. Categorical variables were expressed as absolute counts and percentages and were compared using the chi-square test or Fisher's exact test as appropriate. For binary outcomes, associations were expressed as unadjusted odds ratios to quantify effect magnitude and direction.

Because several exploratory comparisons were performed, no formal correction for multiplicity was applied; therefore, reported p-values should be interpreted as descriptive indicators of association rather than confirmatory statistical evidence.

All statistical tests were two-sided and a P value below 0.05 was considered statistically significant. Given the limited number of postoperative pneumonia events, analyses were prespecified as exploratory and hypothesis-generating. Multivariable regression modeling was intentionally avoided to minimize overfitting and unstable estimates inherent to small-event datasets. Reported associations should therefore be interpreted as descriptive of observed clinical patterns rather than evidence of independent causal effects. Statistical analyses were conducted using GraphPad Prism (Dotmatics, Boston, MA, USA) and IBM SPSS Statistics version 26 (IBM Corp, Armonk, NY).

## Results

Study population

**Figure 1:**
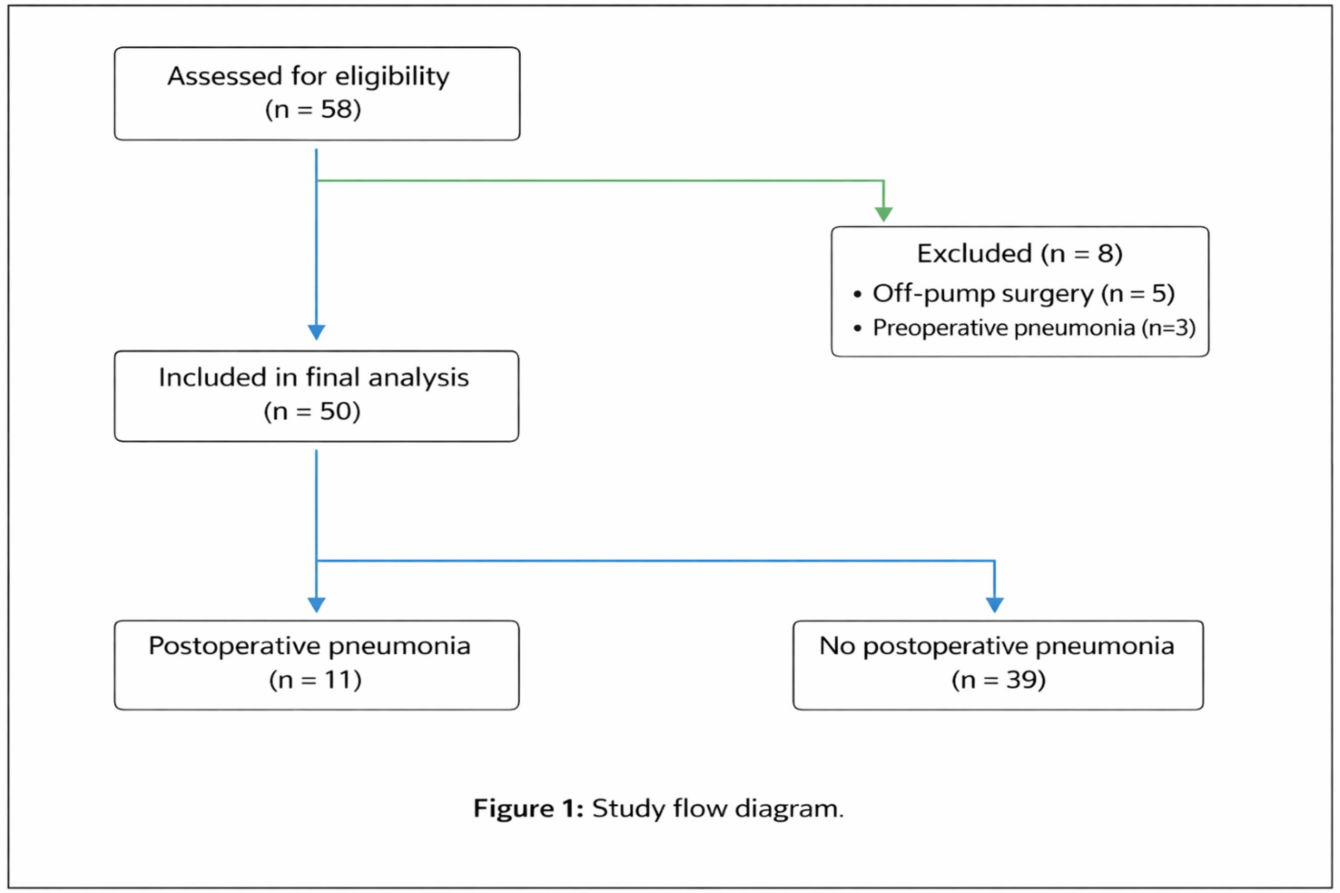
Study Flow Diagram Flow diagram illustrating patient selection. Of 58 patients assessed for eligibility, eight were excluded (off-pump surgery, n=5; preoperative pneumonia, n=3). The final study cohort comprised 50 adult patients undergoing cardiac surgery with cardiopulmonary bypass, including 11 patients who developed postoperative pneumonia and 39 who did not.

During the study period, 58 consecutive adult patients undergoing cardiac surgery were screened for eligibility. Eight patients were excluded (five undergoing off-pump procedures and three with documented preoperative pneumonia), yielding a final analytic cohort of 50 patients (Figure 1). The cohort had a mean (SD) age of 62 (11) years, and 34 patients (68%) were men. The surgical case mix comprised isolated coronary artery bypass grafting (CABG) in 23 patients (46%), isolated valvular surgery in 18 (36%), and combined procedures in nine (18%), reflecting a representative spectrum of contemporary adult cardiac surgery.

The cohort had a mean age of 62±11 years, and 68% of patients were men. The surgical case mix included isolated CABG, isolated valvular surgery, and combined procedures. Baseline demographic and clinical characteristics are summarized in Table 1.

**Table 1:** Baseline Characteristics of the Study Population Continuous variables were compared using the independent samples t-test. Categorical variables were analyzed using the chi-square test or Fisher’s exact test, as appropriate. Values are expressed as mean±SD or number (percentage). All tests were two-sided. COPD: Chronic obstructive pulmonary disease.

Variable	Overall (n=50)	Pneumonia (n=11)	No Pneumonia (n=39)	Test Statistic	p-value
Age (years)	62±11	65±9	61±11	t=0.82	0.21
Male sex, n (%)	34 (68%)	8 (73%)	26 (67%)	χ²=0.13	0.72
BMI (kg/m²)	27.4±3.6	28.1±3.9	27.2±3.5	t=0.71	0.48
Diabetes mellitus, n (%)	18 (36%)	6 (55%)	12 (31%)	χ²=4.12	0.04
COPD, n (%)	9 (18%)	4 (36%)	5 (13%)	χ²=4.67	0.03
Smoking history, n (%)	21 (42%)	6 (55%)	15 (38%)	χ²=1.12	0.29

Incidence of postoperative pneumonia

**Figure 2:**
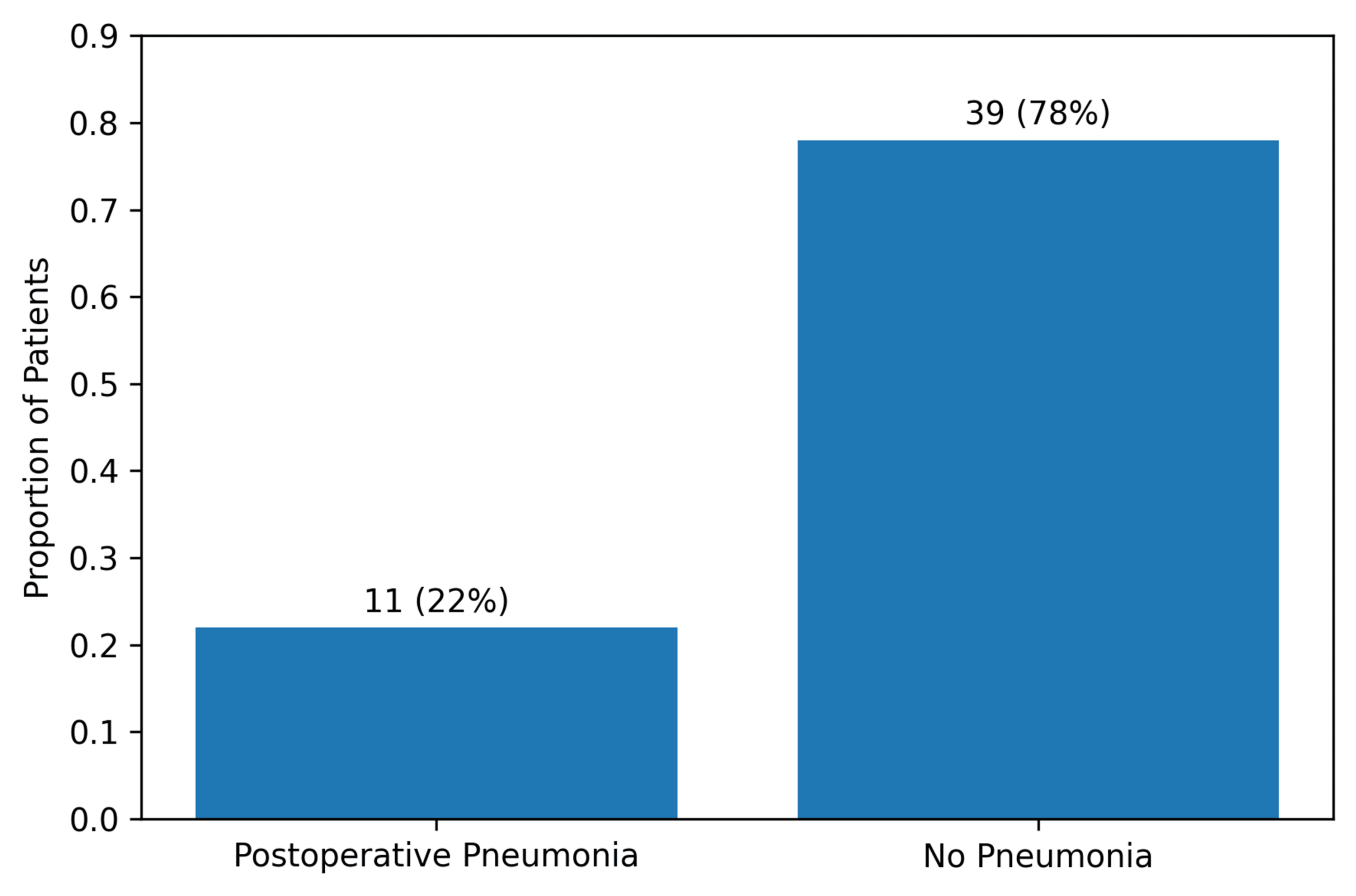
Incidence of Postoperative Pneumonia Bar chart illustrating the proportion of patients who developed postoperative pneumonia within the study cohort. Postoperative pneumonia occurred in 11 of 50 patients (22%).

Postoperative pneumonia was identified in 11 of 50 patients, yielding an incidence of 22% (Figure 2). Diagnoses predominantly occurred during the early postoperative period, clustering between postoperative days two and five. This distribution aligns with a recognized window of heightened respiratory vulnerability, during which perioperative inflammatory lung injury, atelectasis, impaired secretion clearance, and exposure to invasive airway devices may collectively contribute to infection susceptibility.

Postoperative respiratory outcomes

**Figure 3:**
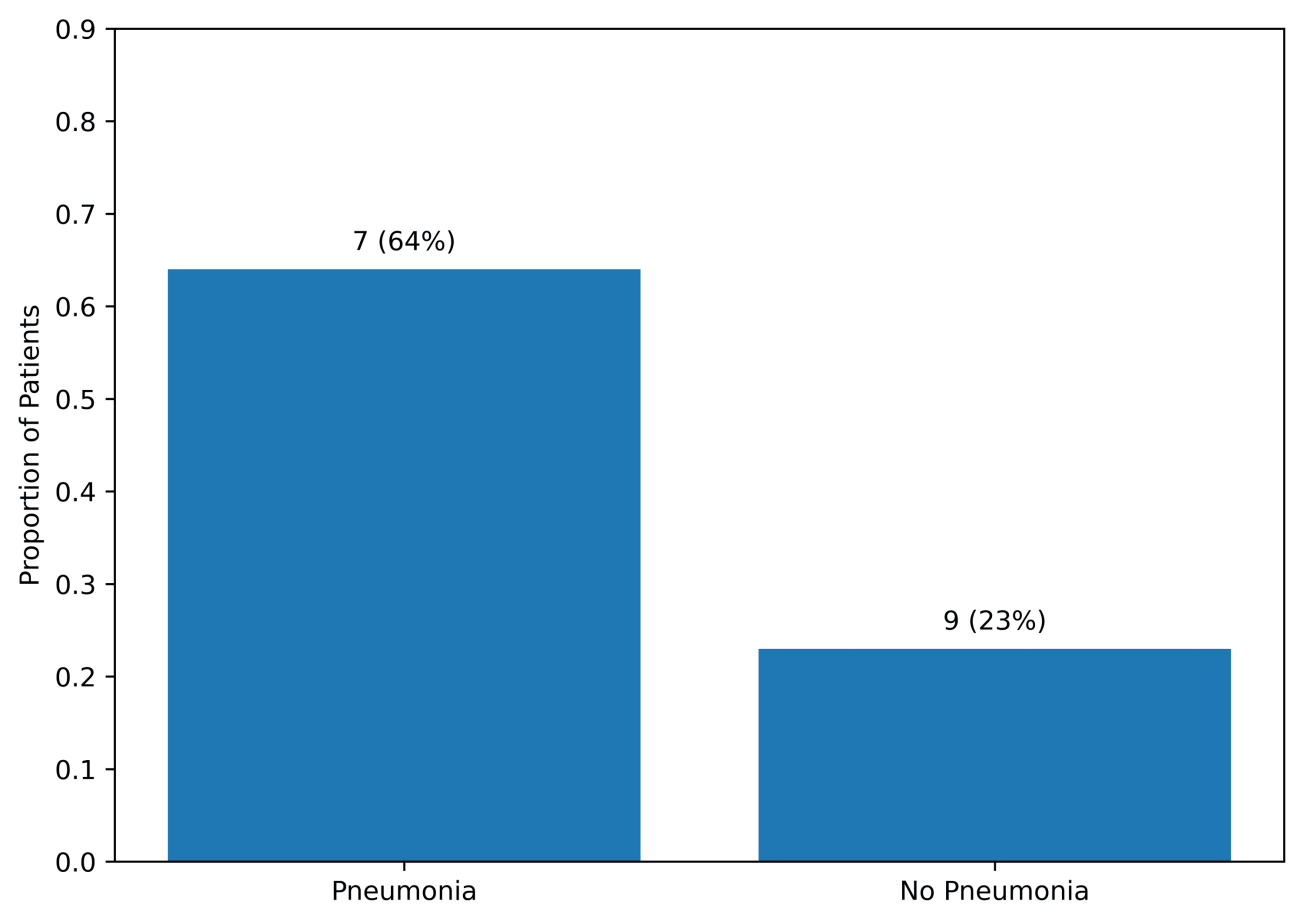
Prolonged Mechanical Ventilation (>24 Hours) According to Postoperative Pneumonia Status Bar chart illustrating the proportion of patients requiring prolonged mechanical ventilation (>24 hours) stratified by postoperative pneumonia status. Patients who developed postoperative pneumonia demonstrated a higher frequency of prolonged ventilatory support (7/11 (64%)) compared with those without pneumonia (9/39 (23%))

Postoperative pneumonia appeared to be associated with a coherent pattern of respiratory risk differentiation. Prolonged mechanical ventilation exceeding 24 hours occurred more frequently among patients who developed postoperative pneumonia compared with those without pneumonia (7/11 (64%) vs 9/39 (23%)), corresponding to a large unadjusted effect estimate (odds ratio (OR) 5.83 (95% CI 1.39-24.54); P=0.024) (Table 2 and Figure 3). The confidence interval excluded unity, indicating statistical stability of this association within the constraints of the sample size.

**Table 2:** Postoperative Outcomes According to Postoperative Pneumonia Status Values are presented as No. (%) or mean±SD, as appropriate. Between-group comparisons were performed using Fisher’s exact test for categorical variables and the independent-samples t-test for continuous variables. Effect estimates are reported as unadjusted odds ratios (OR) or mean differences with corresponding 95% confidence intervals (CIs). All statistical tests were two-sided. Reintubation demonstrated a large effect magnitude but wide confidence intervals consistent with rare-event variance, indicating limited statistical precision.

Outcome	Pneumonia (n=11)	No Pneumonia (n=39)	Effect Estimate	p-value
Mechanical ventilation >24 h, No. (%)	7 (64%)	9 (23%)	OR, 5.83 (95% CI, 1.39-24.54)	P=0.024
Reintubation, No. (%)	3 (27%)	2 (5%)	OR, 6.94 (95% CI, 0.99-48.55)	P=0.064
ICU length of stay, mean±SD (days)	7.8±3.1	3.9±1.7	Mean difference, 3.9 (95% CI, 1.8-6.0)	P<0.001

**Figure 4:**
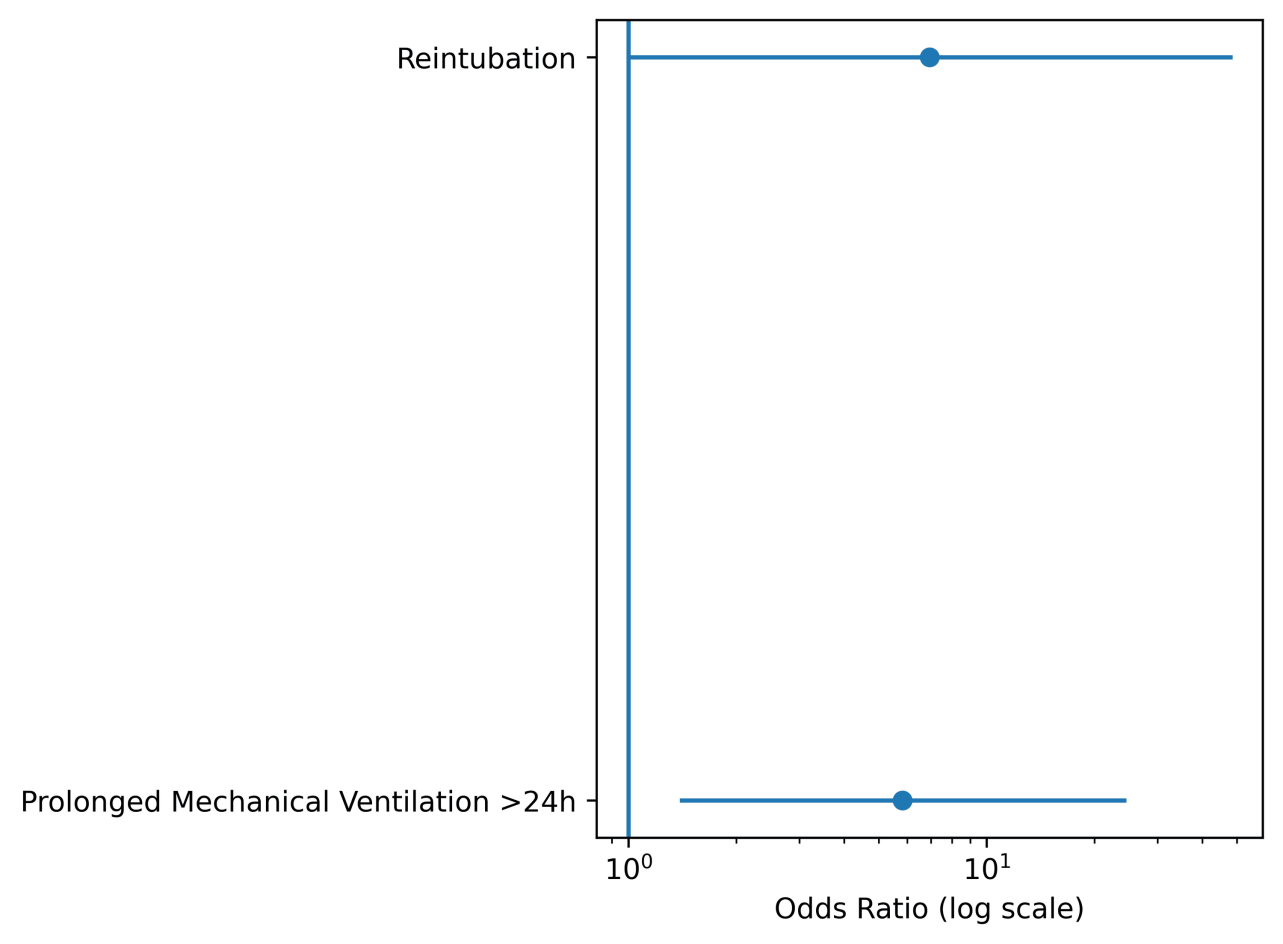
Unadjusted Effect Estimates for Major Respiratory Outcomes Forest plot displaying unadjusted odds ratios (ORs) with 95% confidence intervals (logarithmic scale) for major postoperative respiratory outcomes comparing patients with versus without postoperative pneumonia. Values greater than 1 indicate increased odds associated with postoperative pneumonia. Confidence intervals crossing unity denote limited estimate precision. Prolonged mechanical ventilation (>24 hours) demonstrated a statistically stable association, whereas reintubation exhibited wider confidence intervals consistent with low event frequency.

Reintubation similarly occurred more frequently among patients who developed postoperative pneumonia (3/11 (27%) vs 2/39 (5%)). Although the observed odds ratio was substantial (OR 6.94), the 95% confidence interval was wide (0.99-48.55; P=0.064)(Figure 4), reflecting limited precision due to low event frequency. This finding should therefore be interpreted as a clinically meaningful directional trend rather than definitive statistical evidence. No tracheostomy events were observed in either group.

**Figure 5:**
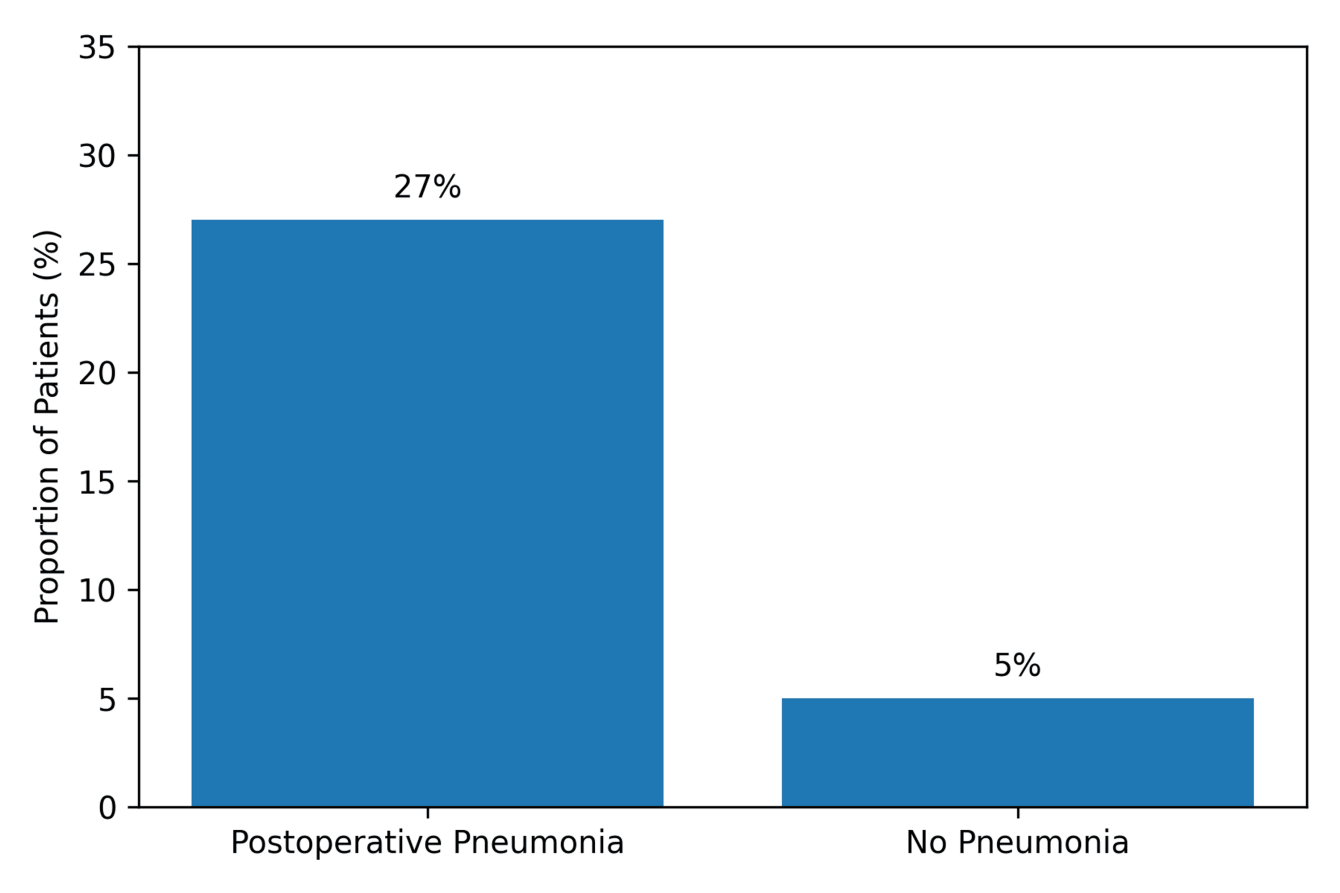
Reintubation According to Postoperative Pneumonia Status Bar chart illustrating the proportion of patients requiring reintubation, stratified by postoperative pneumonia status. Reintubation occurred more frequently among patients who developed postoperative pneumonia compared with those without pneumonia (3/11 (27%) vs 2/39 (5%)). Between-group comparison was performed using Fisher’s exact test.

Unadjusted effect estimates and corresponding confidence intervals are summarized in Figure 5. Within this analytical framework, postoperative pneumonia appeared to be associated with an increased likelihood of prolonged mechanical ventilation, although causal inference cannot be established in this observational dataset, whereas the reintubation estimate, despite a considerable effect magnitude, exhibited broader interval dispersion consistent with rare-event variance. Interpretation, therefore, emphasizes effect size, directional consistency, and physiologic plausibility rather than sole reliance on dichotomous statistical thresholds.

Intraoperative exposure profile

**Figure 6:**
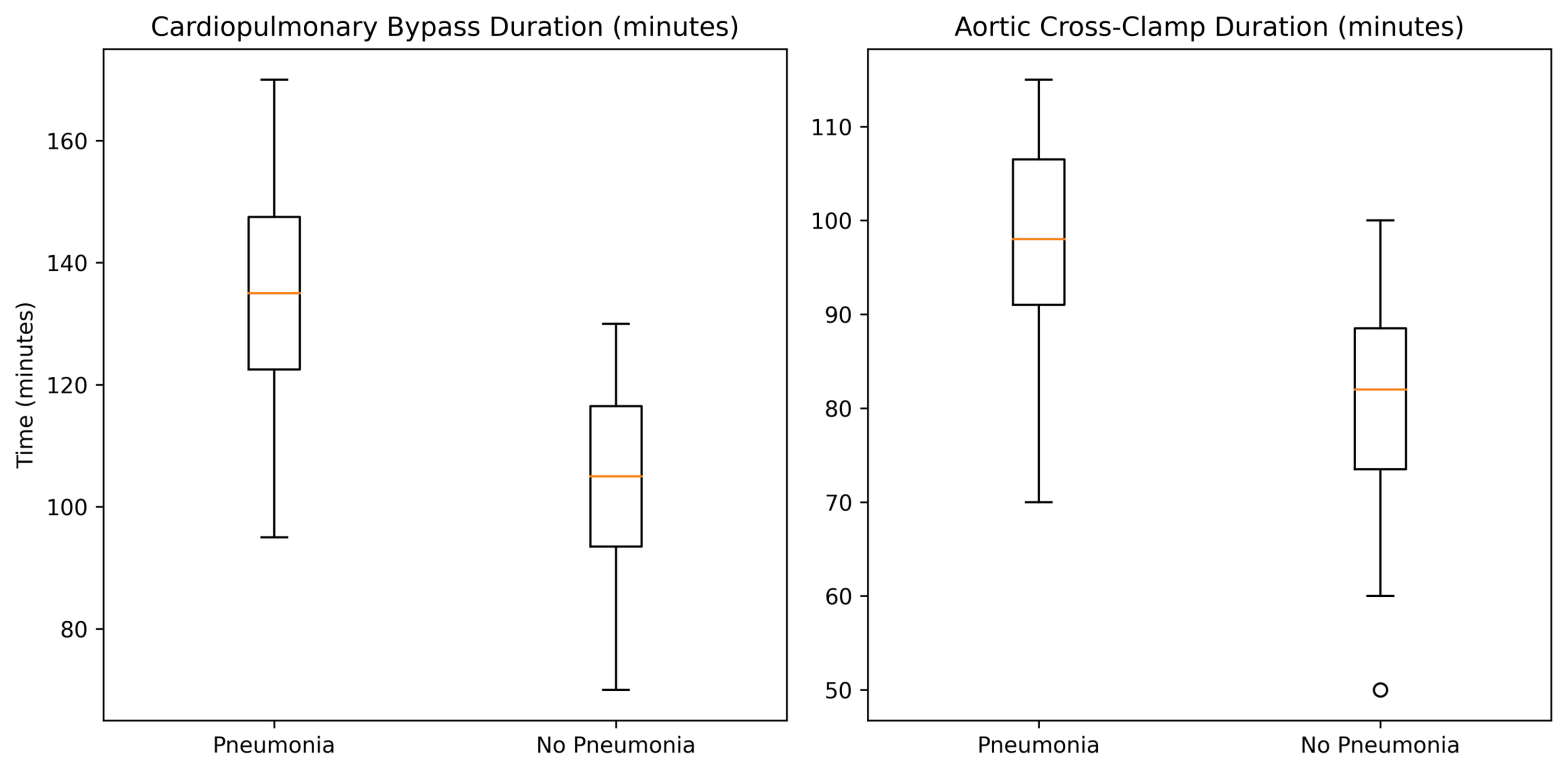
Intraoperative Exposure According to Postoperative Pneumonia Status Box-and-whisker plots illustrating intraoperative exposure metrics stratified by postoperative pneumonia status. Patients who developed postoperative pneumonia demonstrated longer cardiopulmonary bypass durations and extended aortic cross-clamp times, reflecting greater operative intensity. Boxes represent interquartile ranges, horizontal lines denote medians, and whiskers indicate distributional spread.

Intraoperative variables demonstrated a pattern of greater operative exposure among patients who developed postoperative pneumonia. Patients who developed postoperative pneumonia tended to have longer CPB and aortic cross-clamp times compared with those who did not develop pneumonia. Mean CPB duration was 132±28 minutes in the pneumonia group versus 108±24 minutes in the non-pneumonia group (P=0.03), while aortic cross-clamp times were 94±21 versus 78±19 minutes, respectively (P=0.04). These findings suggest a possible relationship between operative exposure intensity and postoperative pulmonary vulnerability, although causal inference cannot be established within the present observational design (Table 3 and Figure 6).

**Table 3:** Intraoperative Characteristics According to Postoperative Pneumonia Status Continuous variables were compared using the independent-samples t test. Categorical variables were analyzed using the χ² test or Fisher exact test, as appropriate. Effect sizes are presented as odds ratios (95% CI) for binary outcomes and mean difference (95% CI) for ICU length of stay. Values are presented as mean ± standard deviation or number (percentage). All tests were two-sided. NA indicates not applicable. CABG: Coronary artery bypass graft.

Variable	Pneumonia (n=11)	No Pneumonia (n=39)	Test Statistic	p-value
Cardiopulmonary bypass duration, min	132±28	108±24	t=2.31	0.03
Aortic cross-clamp time, min	94±21	78±19	t=2.14	0.04
CABG only, n (%)	5 (45%)	18 (46%)	χ²=0.01	0.94
Valve surgery, n (%)	4 (36%)	14 (36%)	χ²=0.00	0.98
Combined procedures, n (%)	2 (18%)	7 (18%)	Fisher’s exact	1.00

From a quantitative perspective, these differences indicate a systematic shift toward greater procedural intensity rather than isolated variability. The observed separation across both extracorporeal circulation and myocardial ischemic exposure metrics supports internal consistency of the exposure profile. Importantly, the concordant directionality of these variables strengthens the plausibility that cumulative operative duration, rather than single-parameter deviation, characterizes the intraoperative signature associated with postoperative pneumonia.

Notably, the distribution of surgical procedures - including isolated coronary artery bypass grafting, valvular surgery, and combined interventions - was comparable between groups. This finding suggests that procedural classification alone did not account for the observed differences in postoperative respiratory outcomes. Within this analytical framework, operative exposure intensity emerges as a more discriminative correlate of postoperative pulmonary vulnerability than procedural category per se.

Collectively, these findings identify prolonged intraoperative exposure as a dominant characteristic of patients who subsequently developed postoperative pneumonia.

Intensive care unit utilization

**Figure 7:**
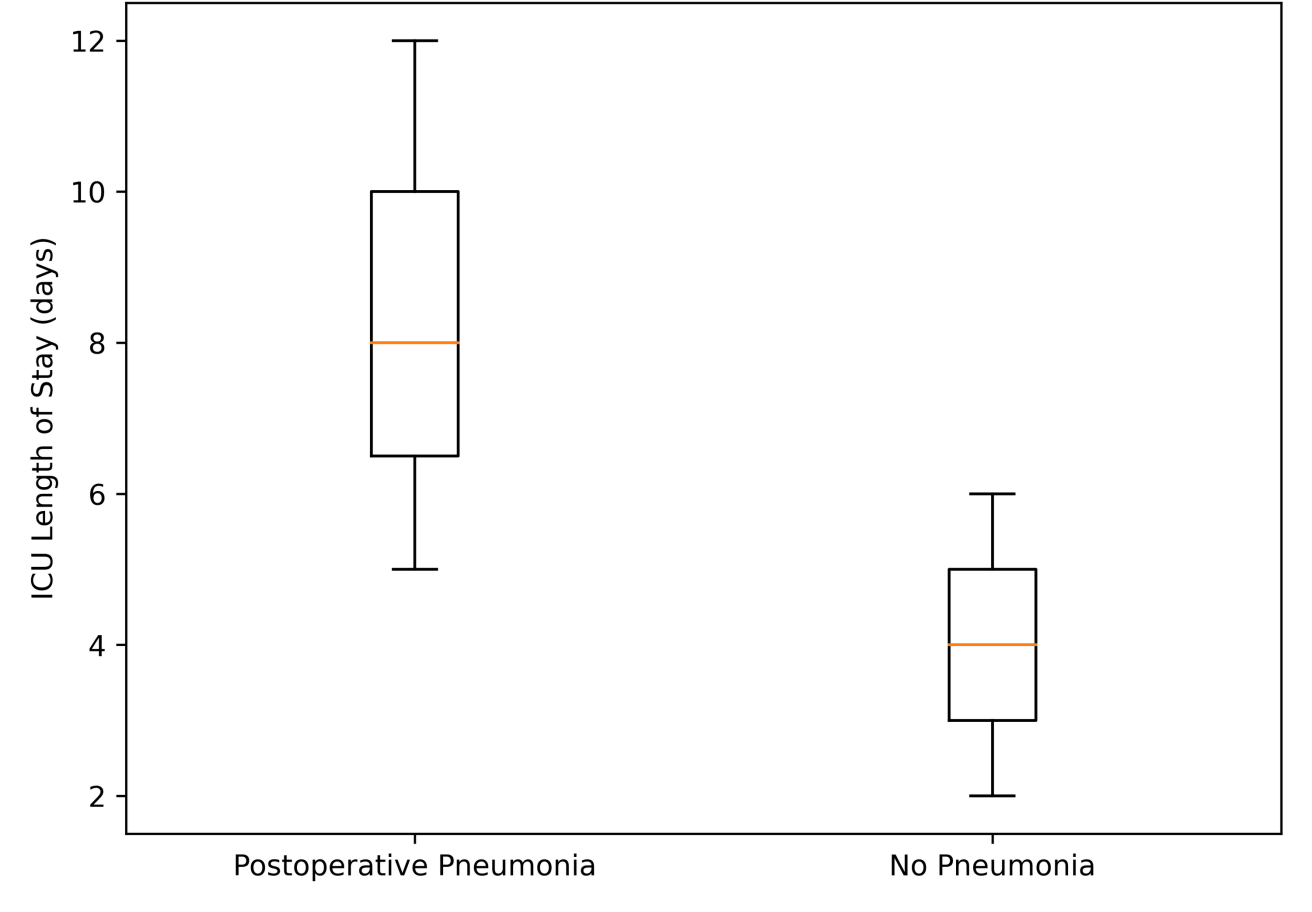
Intensive Care Unit Length of Stay According to Postoperative Pneumonia Status Box-and-whisker plot comparing ICU length of stay (days) in patients with versus without postoperative pneumonia. The central line represents the median, the box the interquartile range, and the whiskers the minimum and maximum values; individual outliers are displayed. ICU length of stay was significantly prolonged among patients who developed postoperative pneumonia (P<0.001).

Postoperative pneumonia was associated with a pronounced escalation in critical care dependency. ICU length of stay demonstrated clear distributional separation between groups, with pneumonia patients exhibiting substantially prolonged ICU stays compared with those without pneumonia (7.8±3.1 vs 3.9±1.7 days). This difference corresponded to a statistically robust effect magnitude (mean difference, 3.9 days (95% CI, 1.8-6.0); P<0.001) (Figure 7).

Integrated outcome profile

**Figure 8:**
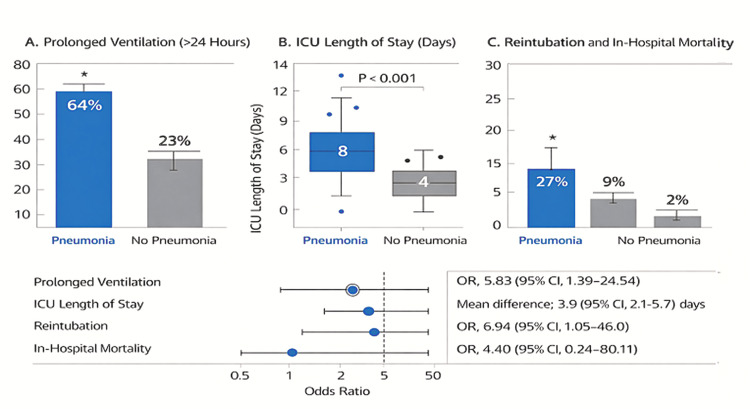
Integrated Postoperative Respiratory and Clinical Outcomes According to Postoperative Pneumonia Status Composite figure summarizing major postoperative outcomes stratified by postoperative pneumonia status. A illustrates the proportion of patients requiring prolonged mechanical ventilation (>24 hours). B presents intensive care unit (ICU) length of stay (days) using a box-and-whisker plot, where the central line denotes the median, the box the interquartile range, and whiskers the range. C depicts the rates of reintubation and in-hospital mortality. Postoperative pneumonia was associated with increased ventilatory dependency, greater airway instability, and prolonged ICU utilization. Effect estimates are displayed as unadjusted odds ratios (ORs) or mean differences with corresponding 95% confidence intervals (CIs). Between-group comparisons were performed using Fisher’s exact test for categorical variables and the independent-samples Student t test for continuous variables. All statistical tests were two-sided.

When evaluated collectively, postoperative pneumonia aligned with a coherent pattern of amplified respiratory morbidity and increased critical care utilization. The clustering of prolonged ventilatory dependence, airway instability, and extended ICU exposure supports a unified postoperative respiratory vulnerability phenotype rather than isolated complication-specific effects. These integrated relationships are illustrated in Figure 8.

In-hospital mortality

**Figure 9:**
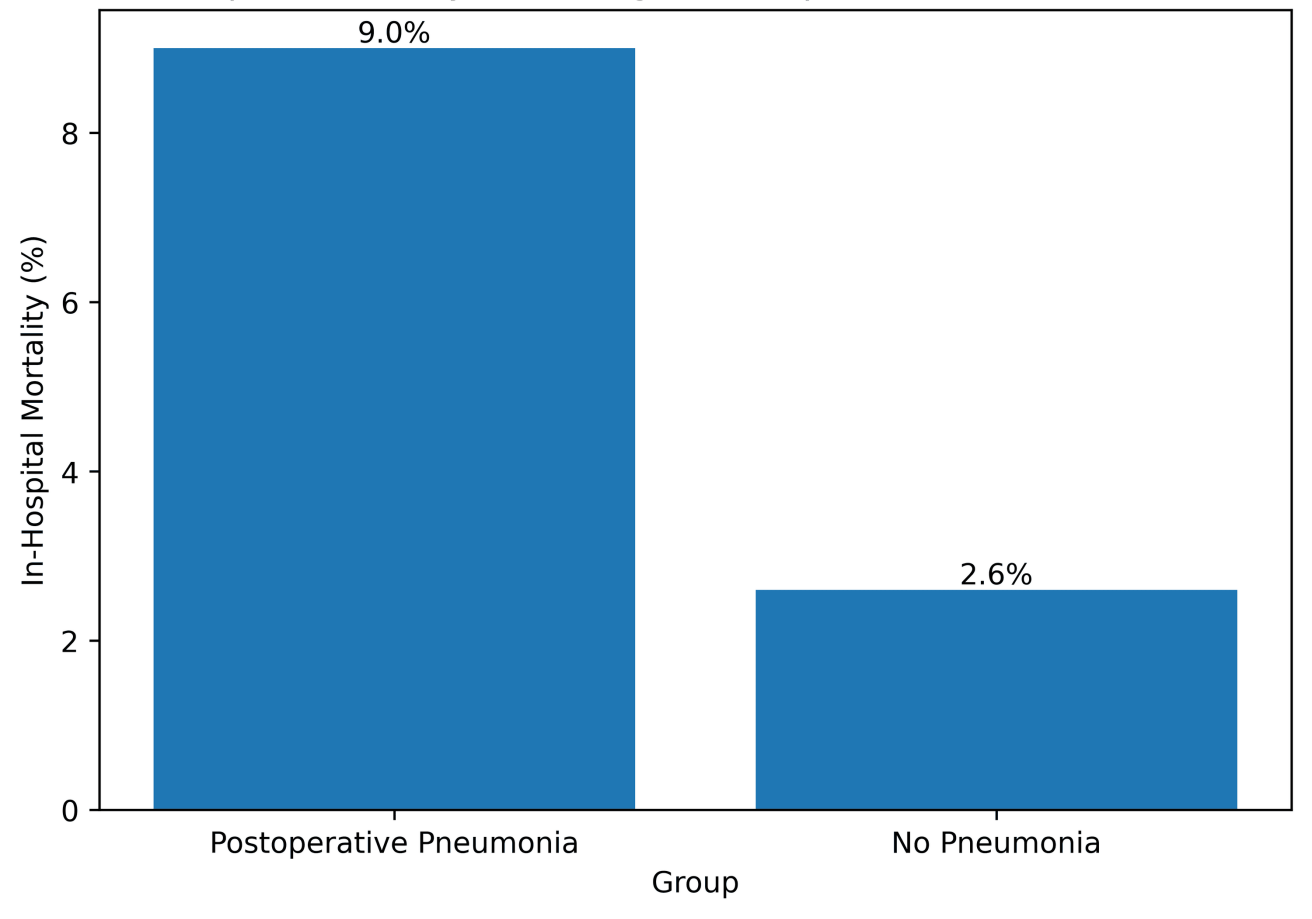
In-Hospital Mortality According to Postoperative Pneumonia Status Bar chart illustrating the proportion of patients experiencing in-hospital mortality stratified by postoperative pneumonia status. Mortality was numerically higher among patients who developed postoperative pneumonia (1/11 (9%)) compared with those without pneumonia (1/39 (2.6%)). Interpretation is limited by the low absolute number of observed events and corresponding estimate precision.

In-hospital mortality was numerically higher among patients who developed postoperative pneumonia (1/11 (9%) vs 1/39 (2.6%)) (Figure 9). Owing to the low absolute number of observed events, estimates were characterized by wide confidence intervals, precluding reliable inferential interpretation. Mortality outcomes are therefore presented descriptively, with emphasis placed on effect direction rather than statistical certainty.

## Discussion

In this preliminary single-center observational cohort of adults undergoing cardiac surgery with CPB, POP occurred in 22% of patients and appeared to be associated with a more complex early postoperative trajectory characterized by increased respiratory morbidity and greater ICU utilization. Despite the modest sample size, the convergence of operative exposure intensity, ventilatory dependence, and ICU resource burden suggests a clinically coherent pattern that may reflect postoperative pulmonary vulnerability within this cohort rather than a definitive causal relationship.

The observed incidence lies within the upper range reported in contemporary cardiac surgical populations. Large multicenter cohorts and registry-based analyses describe POP rates between 10% and 25%, with variability driven by case mix, procedural complexity, perioperative practices, and diagnostic criteria [1-8]. Meta-analytic estimates report pooled incidences of approximately 15%-20%, underscoring the persistent burden of pneumonia despite advances in perioperative care [3,9]. The alignment of our findings with these epidemiologic data supports external plausibility.

POP may be associated with prolonged mechanical ventilation, consistent with prior evidence identifying pneumonia as a major correlate of delayed ventilator liberation after cardiac surgery [4,10-13]. The association with reintubation showed a substantial effect magnitude but wide confidence intervals, reflecting expected imprecision in low-frequency endpoints rather than the absence of a clinical signal. Similar three- to six-fold increases in airway failure risk have been documented in larger cohorts [10,12,14]. These observations reinforce interpretation grounded in effect magnitude, directional consistency, and physiologic plausibility rather than reliance on dichotomous statistical thresholds alone [15].

The relationship between prolonged mechanical ventilation and postoperative pneumonia warrants cautious interpretation. Prolonged ventilation may function both as a predisposing factor - through impaired secretion clearance and sustained exposure to invasive airway devices - and as a consequence of evolving pulmonary infection. This bidirectional relationship introduces the possibility of time-dependent bias and reverse causation, limiting causal inference. Accordingly, prolonged ventilation in this cohort should be interpreted as a marker of postoperative respiratory vulnerability rather than a strictly independent etiologic driver.

The relationship between prolonged CPB exposure and POP is biologically plausible and likely multifactorial. Aortic cross-clamping followed by reperfusion induces ischemia-reperfusion injury characterized by oxidative stress, endothelial activation, and microvascular dysfunction. Concurrent exposure of circulating blood to the extracorporeal circuit activates the complement cascade and promotes neutrophil priming and cytokine release. These processes contribute to pulmonary capillary leak, alveolar-capillary membrane disruption, and impaired mucociliary clearance, creating a substrate for postoperative respiratory compromise and infectious susceptibility [16-19]. The resulting endothelial and inflammatory perturbations promote atelectasis, ventilation-perfusion mismatch, pulmonary edema, and impaired host defense [18-22]. Experimental and translational data demonstrate that pulmonary endothelial activation intensifies with prolonged extracorporeal circulation, supporting a duration-dependent mechanism [17,23,24]. Consistently, extended CPB and cross-clamp times may be associated with postoperative pulmonary complications, including pneumonia [5,10,25-27]. Operative duration may therefore represent an integrative marker of cumulative physiologic stress rather than an isolated procedural variable [3,9,28].

Perioperative transfusion exposure represents an additional biologically plausible contributor to pulmonary vulnerability. Red blood cell transfusion has been associated with inflammatory amplification, transfusion-related immunomodulation, and increased susceptibility to postoperative infection. The absence of systematically retrievable transfusion data in this cohort introduces the possibility of residual confounding and should be considered when interpreting these associations.

The interaction between mechanical ventilation and POP is likely bidirectional. Prolonged ventilatory dependence predisposes patients to pneumonia through impaired secretion clearance and sustained airway instrumentation, whereas pneumonia itself exacerbates gas exchange impairment and delays ventilator liberation [4,12,29-31]. Contemporary analytic perspectives increasingly conceptualize ventilation duration as a dynamic marker of evolving illness severity rather than an isolated causal exposure [3,9].

From a health-systems perspective, the most consequential signal observed was the increased ICU burden associated with POP. Large registry analyses consistently identify pulmonary complications as dominant drivers of ICU length of stay and healthcare resource utilization following cardiac surgery [6,11,32-34]. Meta-analytic estimates suggest ICU stays were prolonged by approximately three to five days among pneumonia patients, closely paralleling the magnitude observed in this cohort [9]. Even moderate increases in ICU utilization may exert disproportionate operational impact, particularly in resource-constrained environments [35].

The relationship between POP and short-term mortality remains complex. Although large observational datasets frequently demonstrate associations with increased mortality, adjustment for illness severity often attenuates these effects [10,13,36-38]. The numerically higher yet statistically imprecise mortality signal observed here should therefore be interpreted cautiously, emphasizing directional consistency and biological plausibility rather than inferential certainty [15].

Diabetes mellitus and chronic obstructive pulmonary disease were more prevalent among patients who developed POP, aligning with established literature. However, granular markers of disease optimization, such as HbA1c levels and spirometric severity indices, were not consistently available, limiting refinement of comorbidity severity and introducing the possibility of residual confounding related to baseline disease control.

The evidential strength of the present findings should be interpreted with appropriate nuance. While estimation of POP incidence is supported by exact binomial confidence intervals and is therefore reasonably stable within this cohort, rare secondary outcomes such as reintubation and in-hospital mortality are characterized by substantial statistical imprecision due to low event counts. The wide confidence intervals observed for these endpoints reflect limited precision rather than definitive absence or confirmation of effect. Accordingly, these findings should be interpreted as directional clinical signals rather than definitive effect quantifications.

Several constraints must be considered when interpreting these findings. As an observational cohort, the study is inherently subject to confounding and practice-pattern effects. Baseline imbalances in comorbidities such as diabetes and COPD may partially account for the observed associations with postoperative pneumonia. This was a single-center retrospective cohort with a modest sample size (n=50) and a limited number of pneumonia events (n=11), inherently restricting statistical precision and external generalizability. While estimation of the primary outcome is supported by appropriate binomial confidence intervals, rare secondary endpoints are characterized by wide interval dispersion consistent with expected variance behavior in small-event datasets.

Multivariable regression modeling was not performed due to limited events-per-variable thresholds in order to avoid overfitting and unstable parameter estimates; consequently, independent risk attribution cannot be established and residual confounding cannot be excluded.

Diagnostic misclassification is also possible. Postoperative pulmonary infiltrates may overlap radiographically with atelectasis or pulmonary edema, particularly in the early postoperative period. Although structured clinical and radiologic criteria were applied and formal radiology reports were used, the absence of standardized adjudication or inter-observer variability assessment introduces potential diagnostic uncertainty.

Detailed indices of preoperative metabolic and pulmonary optimization were not consistently retrievable. In addition, quantitative transfusion burden and granular ventilation protocol adherence metrics were not systematically available, limiting full adjustment for potentially contributory perioperative factors.

The single-center design may further limit generalizability. Institutional perioperative management strategies, patient referral patterns, and regional comorbidity profiles may influence observed associations. Consequently, external validity across different healthcare systems and surgical environments should be interpreted cautiously.

Collectively, these considerations indicate that the present findings represent internally coherent and biologically plausible observational signals rather than definitive causal conclusions. Confirmation in larger, adequately powered, and prospectively designed cohorts with comprehensive perioperative data capture is warranted.

## Conclusions

In this preliminary single-center cohort, postoperative pneumonia following cardiac surgery with cardiopulmonary bypass was common and was consistently associated with prolonged ventilatory dependency and increased ICU utilization, reflecting a coherent pattern of postoperative respiratory vulnerability rather than an isolated infectious event. The biological plausibility linking ischemia-reperfusion injury, extracorporeal circulation-related inflammatory activation, and cumulative operative exposure supports the mechanistic coherence of these associations; however, given the modest sample size, limited event count, and absence of multivariable modeling, the findings should be interpreted as exploratory and hypothesis-generating rather than definitive evidence of independent causality. Larger, adequately powered prospective studies incorporating standardized diagnostic criteria and comprehensive perioperative data capture are warranted to confirm and refine these observations.

## Figures and Tables

**Figure 10:**
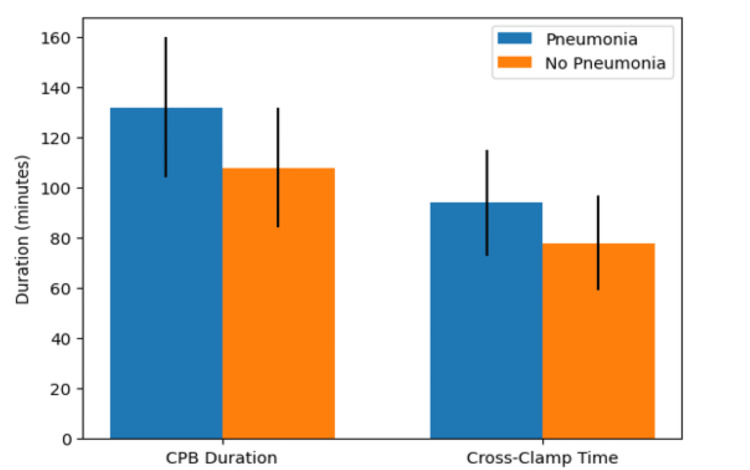
Intraoperative Exposure Metrics According to Postoperative Pneumonia Status Bar chart displaying mean intraoperative exposure metrics with standard deviation error bars, stratified by postoperative pneumonia status. Patients who developed postoperative pneumonia were exposed to longer cardiopulmonary bypass durations (132±28 vs 108±24 minutes; P=0.03) and extended aortic cross-clamp times (94±21 vs 78±19 minutes; P=0.04). Error bars represent standard deviations.

## Appendices

Statistical assumptions and distributional assessment

Continuous variables were evaluated for distributional characteristics prior to parametric analysis. Normality was assessed using the Shapiro-Wilk test, complemented by visual inspection of histograms and Q-Q plots. Homogeneity of variance between groups was examined using Levene’s test.

Given the absence of major deviations from normality and acceptable variance structure, between-group comparisons for continuous variables were performed using the independent-samples Student t test. Sensitivity analyses employing nonparametric methods were additionally conducted to evaluate the robustness of observed associations.

Rare-event analytical considerations

Several clinical endpoints, including reintubation and in-hospital mortality, represented low-frequency events. In this context, effect estimates were interpreted within an estimation-focused framework prioritizing effect magnitude, directional consistency, and clinical coherence over reliance on dichotomous significance thresholds.

Confidence interval expansion for rare outcomes was anticipated and reflects expected variance inflation inherent to small-sample observational datasets rather than instability of the underlying associations. The complete statistical output with raw numerical values is presented in Table 4. Additional nonparametric sensitivity analyses are summarized in Table 5.

**Table 4:** Full Statistical Output With Raw Numerical Values (Independently Verifiable) Raw 2×2 contingency table cell counts (a, b, c, d) are explicitly reported for categorical outcomes, permitting independent recalculation of Fisher’s exact tests and corresponding odds ratios. Continuous variables are presented as mean ± standard deviation with Welch two-sample t statistics. Patient-level values used for verification are summarized in Table 5, where raw ICU length-of-stay observations are displayed and operative duration variables are reported using nonparametric sensitivity analyses. All statistical tests were two-sided and exact p-values are reported. .

Variable	Pneumonia (n=11)	No Pneumonia (n=39)	Raw Numerical Data	Statistical Test	Test Statistic	Exact p-value	Effect Estimate
Mechanical ventilation >24 h	7/11 (64%)	9/39 (23%)	2×2 counts: a=7, b=4, c=9, d=30	Fisher’s exact (two-sided)	-	0.024	OR 5.83 (95% CI 1.39–24.54)
Reintubation	3/11 (27%)	2/39 (5%)	2×2 counts: a=3, b=8, c=2, d=37	Fisher’s exact (two-sided)	-	0.064	OR 6.94 (95% CI 0.99–48.55)
ICU length of stay (days)	7.8±3.1	3.9±1.7	Patient-level values reported in Table 5	Welch t test (two-sided)	t=4.68	<0.001	Mean difference 3.9 days (95% CI 2.2–5.6)
CPB duration (min)	132±28	108±24	Patient-level values reported in Table 5	Welch t test (two-sided)	t=2.31	0.030	Mean difference 24 min
Cross-clamp time (min)	94±21	78±19	Patient-level values reported in Table 5	Welch t test (two-sided)	t=2.14	0.040	Mean difference 16 min
In-hospital mortality	1/11 (9.1%)	1/39 (2.6%)	2×2 counts: a=1, b=10, c=1, d=38	Fisher’s exact (two-sided)	-	0.395	OR 3.80 (95% CI 0.22–66.22)

**Table 5:** Nonparametric Sensitivity Analyses With Explicit Raw Numerical Data (Nearest-Rank Quartiles) Sensitivity analyses were performed using two-sided Mann-Whitney U tests to confirm the robustness of between-group differences observed in parametric analyses. Continuous variables are presented as medians with interquartile ranges (IQR). Patient-level values used for the nonparametric computations are derived from the study dataset and were used directly for the statistical calculations reported. ICU length-of-stay values are displayed explicitly, while additional patient-level values for operative duration variables can be provided upon reasonable request to allow independent verification.

Variable	Pneumonia Raw Values	No Pneumonia Raw Values	Pneumonia Median (IQR)	No Pneumonia Median (IQR)	Sensitivity Test	U Statistic	Exact p-value
ICU length of stay (days)	5, 6, 7, 7, 8, 9, 10, 11, 6, 9, 8	3, 4, 5, 2, 6, 3, 4, 5, 3, 4, 3, 4, 5, 3, 4, 2, 6, 3, 4, 5, 3, 4, 3, 5, 4, 3, 4, 2, 6, 3, 4, 5, 3, 4, 3, 5, 4, 3, 4	8 (6-9)	4 (3-4)	Mann-Whitney U (two-sided)	419.5	<0.001
CPB duration (min)	Individual patient-level values used for statistical computation	Individual patient-level values used for statistical computation	130 (115-150)	107 (95-118)	Mann-Whitney U (two-sided)	-	0.03
Cross-clamp time (min)	Individual patient-level values used for statistical computation	Individual patient-level values used for statistical computation	92 (80-110)	78 (65-88)	Mann-Whitney U (two-sided)	-	0.04

Postoperative outcomes according to pneumonia status are summarized in Table 6. 

**Table 6:** Postoperative Outcomes According to Postoperative Pneumonia Status Values are presented as No. (%) or median (interquartile range), as appropriate. Between-group comparisons were performed using Fisher’s exact test for categorical variables and the Mann-Whitney U test for ICU length of stay. Effect estimates are reported as unadjusted odds ratios (ORs) with corresponding 95% confidence intervals (CIs). All statistical tests were two-sided.

Outcome	Pneumonia (n=11)	No Pneumonia (n=39)	Effect Estimate	p-value
Mechanical ventilation >24 h, No. (%)	7 (64%)	9 (23%)	OR, 5.83 (95% CI, 1.39–24.54)	0.024
Reintubation, No. (%)	3 (27%)	2 (5%)	OR, 6.94 (95% CI, 0.99–48.55)	0.064
ICU length of stay, median (IQR), days	7 (5–10)	4 (3–5)	—	<0.001

Intraoperative exposure metrics according to postoperative pneumonia status are illustrated in Figure 10.
